# Anti-cancer and anti-inflammatory effects elicited by short chain fatty acids produced by *Escherichia coli* isolated from healthy human gut microbiota

**DOI:** 10.1186/s12934-020-01477-z

**Published:** 2021-02-05

**Authors:** Atchareeya Nakkarach, Hooi Ling Foo, Adelene Ai-Lian Song, Nur Elina Abdul Mutalib, Sunee Nitisinprasert, Ulaiwan Withayagiat

**Affiliations:** 1 Department of Bioprocess Technology, Faculty of Biotechnology and Biomolecular Sciences, Universiti Putra Malaysia, 43400 UPM Serdang, Selangor Malaysia; 2 Department of Biotechnology, Faculty of Agro-Industry, Kasetsart University, Chatuchak, 10900 Bangkok Thailand; 3 Institute of Bioscience, Universiti Putra Malaysia, 43400 UPM Serdang, Selangor Malaysia; 4 Department of Microbiology, Faculty of Biotechnology and Biomolecular Sciences, Universiti Putra Malaysia, 43400 UPM Serdang, Selangor Malaysia; 5 Agro-Biotechnology Institute, National Institutes of Biotechnology Malaysia, 43000 UPM Serdang, Selangor Malaysia; 6 Fermentation Technology Research Center, Faculty of Agro‑Industry, Kasetsart University, Chatuchak, 10900 Bangkok Thailand

**Keywords:** Anti-cancer, Anti-inflammatory, Cytokines, *Escherichia coli*, Short chain fatty acid

## Abstract

**Background:**

Extracellular metabolites of short chain fatty acids (SCFA) excreted by gut microbiota have been reported to play an important role in the regulation of intestinal homeostasis. Apart from supplying energy, SCFA also elicit immune stimulation in animal and human cells. Therefore, an attempt was conducted to isolate SCFA producing bacteria from healthy human microbiota. The anti-cancer and anti-inflammatory effects of extracellular metabolites and individual SFCA were further investigated by using breast, colon cancer and macrophage cells. Toxin, inflammatory and anti-inflammatory cytokine gene expressions were investigated by RT-qPCR analyses in this study.

**Results:**

*Escherichia coli* KUB-36 was selected in this study since it has the capability to produce seven SCFA extracellularly. It produced acetic acid as the main SCFA. It is a non-exotoxin producer and hence, it is a safe gut microbiota. The IC_50_ values indicated that the *E. coli* KUB-36 metabolites treatment elicited more potent cytotoxicity effect on MCF7 breast cancer cell as compared to colon cancer and leukemia cancer cells but exhibited little cytotoxic effects on normal breast cell. Furthermore, *E. coli* KUB-36 metabolites and individual SCFA could affect inflammatory responses in lipopolysaccharide-induced THP-1 macrophage cells since they suppressed inflammatory cytokines IL-1β, IL-6, IL-8 and TNF-α well as compared to the control, whilst inducing anti-inflammatory cytokine IL-10 expression.

**Conclusion:**

SCFA producing *E. coli* KUB-36 possessed vast potential as a beneficial gut microbe since it is a non-exotoxin producer that exhibited beneficial cytotoxic effects on cancer cells and elicited anti-inflammatory activity simultaneously. However, the probiotic characteristic of *E. coli* KUB-36 should be further elucidated using in vivo animal models.
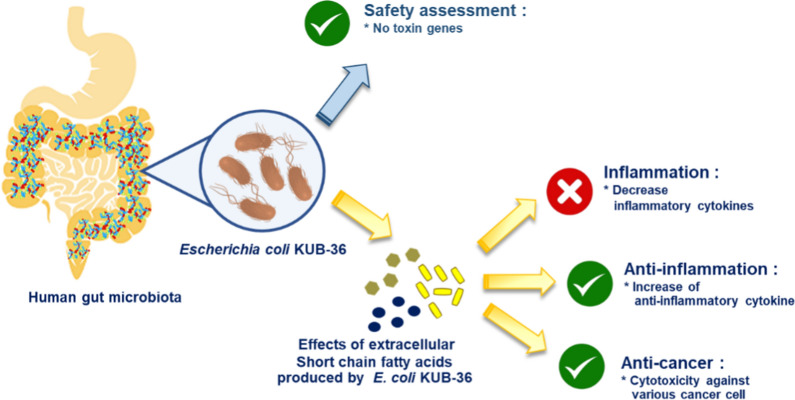

## Background

Gut microbiota is a large and diverse group of microorganisms that live in the gastrointestinal tract. The human gut contains approximately 10^14^ microorganisms, which play an important role in gut homeostasis which impact host metabolism, health and disease [1, 2]. One of the important metabolites that is produced by gut microbiota is short chain fatty acid (SCFA). The major SCFA are acetic, butyric and propionic (≥ 95%) acids, whereas formic, valeric, caproic acids, etc., make up the remaining SCFA [3], whereby they reduce the luminal pH, enhance the absorption of some nutrients and have direct impact on the gut microbiota composition [4]. Moreover, SCFA are the main energy source for colon cells. It has been demonstrated that 70% of energy obtained by intestinal epithelial cells is derived from butyric acid, which is mainly produced by commensal bacteria, especially from the Firmicutes phyla such as *Ruminococcus* and *Faecalibacterium* [5]. However, *Escherichia coli* Nissle 1917, a Gram-negative bacterium that has been reported to be a probiotic bacterium also produces SCFA as the end products of carbohydrate metabolism under anaerobic growth condition, with mainly acetic acid and some propionic and butyric acids [6].

SCFA has been shown to have an impact on the immune system, which can reduce the risk of inflammatory diseases, type 2 diabetes, obesity, heart disease and other symptoms including cancer [7]. Chronic inflammation induces persistent damage and plays a role in the development of cancer [8]. Cancer is a leading cause of death worldwide, especially breast cancer and colorectal cancer [9]. Nowadays, chemoprevention is considered generally as a better means than the therapeutic approach. Probiotic supplement has been used as biotherapeutic and have been reported to have anti-cancer effect [10], whereby the beneficial effects of probiotic bacteria have been regarded to be mediated by their extracellular metabolites of SCFA.

Moreover, SCFA have been demonstrated to modulate the production of inflammatory mediators by macrophages. SCFA, especially butyric acid, suppress the productions of lipopolysaccharide (LPS), nitric oxide (NO) and common cytokines such as tumor necrosis factor α (TNF-α), interleukins 6 and 8 (IL-6, and IL-8), which are known to induce inflammatory responses. In addition, SCFA also enhance the expression of anti-inflammatory cytokine such as Interleukins (IL-10) [11–16]. *Faecalibacterium prausnitzii* is the first anti-inflammatory commensal bacteria that was identified based on human clinical data and it is one of the major butyric acid-producer of the human intestinal microbiota [17, 18]. However, other gut microbiota such as *Bifidobacterium* and *Lactobacillus* species have been reported to exhibit anti-inflammatory property [19–22], along with *E. coli* Nissle 1917, which is a commensal bacterium with defect in its LPS biosynthesis and hence it is accepted and used as a probiotic strain for the treatment of inflammatory gastrointestinal diseases [23]. Interestingly, Tsilingiri et al. [24] has proposed the utilisation of postbiotic metabolites produced by the probiotic *Lactobacillus* sp. as a safe alternative for the treatment of patients with inflammatory bowel disease in the acute inflammatory phase. Subsequently, prominent probiotic effects of bacteriocin-containing postbiotic metabolites produced by six strains of *Lactobacillus plantarum* have been documented extensively for rats [25–27], poultry [28, 29], pigs [30] and lambs [31]. The *L. plantarum* strains produce several postbiotic metabolites such as lactic acid, acetic acid [32, 33] and bacteriocin [34, 35]. Recently, postbiotic metabolites produced by the six strains of *L. plantarum* have been shown to elicit selective cytotoxic effect and induction of apoptosis via a strain-specific and cancer cell type-specific manner whilst sparing the normal cells [36]. Therefore, the postbiotic metabolites of *L. plantarum* were suggested as a functional supplement and as an adjunctive treatment for cancer.

In the present study, an attempt was conducted to characterize the SCFA produced by a beneficial, *Escherichia coli* KUB-36 that was isolated from healthy human gut microbiota. The anti-cancer effects of the SCFA-consisting metabolite of *E. coli* KUB-36 were subsequently determined by using normal cell line, breast cancer cell line, colon cancer cell line and leukemia cancer cell line. Moreover, the anti-inflammatory effect of the *E. coli* KUB-36 metabolite were elucidated under inflammatory condition using a gene expression approach. The individual SCFA that produced by *E. coli* KUB-36 were also investigated simultaneously to characterize the main SCFA that elicited the anti-cancer and anti-inflammatory activities.

## Results

### Isolation and characterization of SCFA producing gut microbiota

**Fig. 1:**
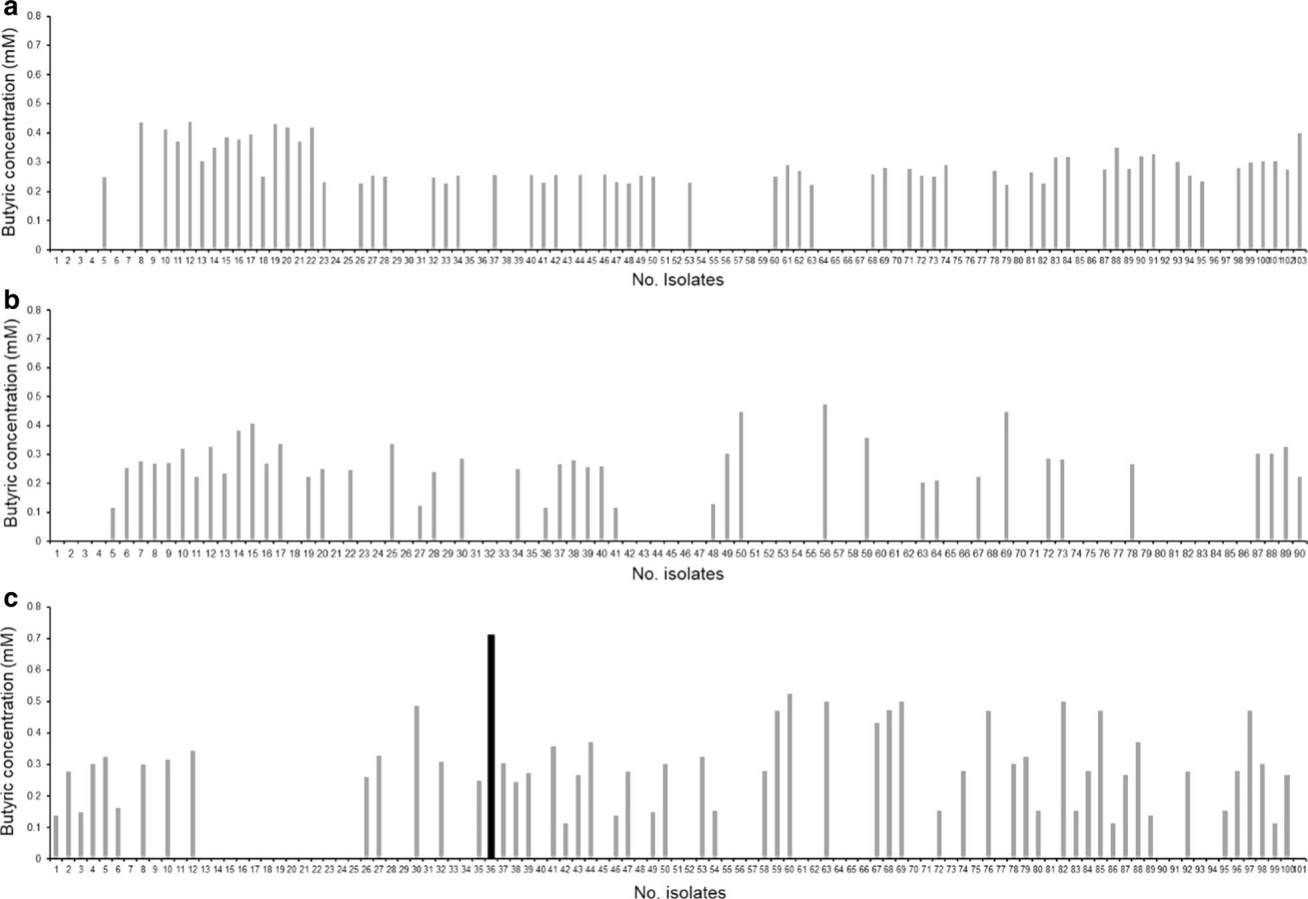
Butyric acid concentration of 294 bacterial isolated from 3 heathy human faeces. **a** Butyric acid concentration of 103 bacteria isolated from faecal sample 1. **b** Butyric acid concentration of 90 bacteria isolated from faecal sample 2 and **c** butyric acid concentration of 101 bacteria isolated from faecal sample 3. Black bar chart represents bacterial isolate producing the highest butyric acid

A total of 294 bacterial cultures were isolated from 3 feces samples of healthy Thai adults. The acid producing bacteria was then screened preliminarily by a change of color in bromothymol blue dye. The extracellular SCFA profile of 185 acid producing bacteria isolates were distinguished subsequently by gas chromatography. The highest butyric acid producing bacteria were selected for phenotypic and genotypic identification and characterization (Fig. 1). The morphological observation via Gram staining showed that the isolated bacteria was a Gram‐negative and rod shaped bacterial. It was then designated as *E. coli* KUB-36 since the alignment of the 16S rRNA gene demonstrated more than 99% sequence similarity to the NCBI deposited 16S rRNA genes of *E. coli* strains (results not shown). The threshold of 98.7% similarity for 16S rRNA genes is essential for species identification [37]. To confirm the potential health benefits as a probiotic bacterium, *E. coli* KUB-36 was subsequently elucidated for anti-inflammatory and anti-cancer properties.

### Toxin genes detection

Lipopolysaccharide (LPS) is a large molecule found on the outer membrane of Gram-negative bacteria that generally elicits strong immune response in humans and animals. Hence, the presence of 10 LPS endotoxin genes and two exotoxin genes in *E. coli* KUB-36 were determined by PCR in this study using specific primers that were designed based on the conserved regions analysed by multiple alignments of DNA sequences deposited in the NCBI database. Table 1 shows the primer sequences and toxin gene profiles detected for *E. coli* KUB-36. The results showed that *E. coli* KUB-36 was positive for *lpxA, lpxB, lpxC, lpxD, waaF* and *wzy* endotoxin genes, but negative for the *waaC, waaL, waaQ, wzz* endotoxin genes and both *eltB* and *stb* exotoxin genes. These results indicated that *E. coli* KUB-36 did not harbour endotoxin genes that responsible for LPS biosynthesis, hence, resulting in a defective LPS production in *E. coli* KUB-36.

**Table 1:** Endotoxin and exotoxin gene profiles of *E. coli* KUB-36

Toxin	Genes	Sequence	Products Size (bp)	Toxin gene detection	
				*E. coli* KUB-36	Pathogenic *E. coli*
Endotoxin	*IpxA*	5′GATACGTGATTGATAAATCC3′	799	+	+
		3′AGTCATTAACGAATCAGACC5′			
	*lpxB*	5′CGTTAATGACTGAACAGCGT3′	1159	+	+
		3′TTCGATCATTGTGCTAACTC5′			
	*lpxC*	5′ATACGATGATCAAACAAAGG3′	928	+	+
		3′TGTCGTTATGCCAGTACAGC5′			
	*lpxD*	5′AAGTAATGCCTTCAATTCGA3′	1036	+	+
		3′GAACGTTAGTCTTGTTGATT5′			
	*waaC*	5′GACGGATGCGGGTTTTGATC3′	970	−	+
		3′TACCTTTATAATGATGATAA5′			
	*waaF*	5′TCTGCATGAAAATACTGGTG	1057	+	+
		3′ATCCGTCAGGCTTCCTCTTG5′			
	*waaL*	5′AAAACATGCTAACATCCTTT3′	1270	−	+
		3′TATTCTTAATTAATTGTATT5′			
	*waaQ*	5′CGGTGCTAGTATTAACACGT3′	1043	−	+
		3′ATTTCTACGCTATAGTACCC5′			
	*wzy*	5′GTCGCATGAGTCTGCTGCAA3′	1363	+	+
		3′CATTGTTATCCTTCAACCTG5′			
	*wzz*	5′CTTTTCTAATGAAGCGCAAC3′	988	−	+
		3′ATTACTACTCTCATCTTTTA5′			
Enterotoxin	*etlB*	5′GTTGACATATATAACAGAATTCGGGATGAA3′	604	−	+
		3′AAGCTTGCCCCTCCAGCCTAGCTTAGTTTT5′			
	*Stb*	5′TATTATATTTCGAAGCTTAAGTATTGTTGA3′	1087	−	+
		3′CATGACACGAAGCGCAGGCTGTTGCGCACC5′			

+ : present; − : not present

### Short chain fatty acid profile

*E. coli* KUB-36 was grown in Wilkins Chalgren broth at 37 °C for 24 h under anaerobic condition, whereby the SCFA were analysed at four-hour intervals by gas chromatography. Table 2 shows the SCFA concentrations produced by *E. coli* KUB-36. The highest SCFA was detected at 8 h of incubation time. Interestingly, the selected *E. coli* KUB-36 produced mainly acetic acid (23.89 mM), followed by caproic acid, butyric acid, valeric acid, iso-valeric acid, propionic acid and iso-butyric acid with 3.63, 3.01, 2.83, 2.78, 2.15 and 1.69 mM respectively. Therefore, the concentrations of SCFA at 8 h of incubation was used for subsequent study.

**Table 2:** Short chain fatty acid profiles produced by *E. coli* KUB-36

SCFA	SCFA concentration (mM)					
	4 h	8 h	12 h	16 h	20 h	24 h
Acetic acid	21.45 ± 0.01^d^	23.89 ± 0.12^a^	23.47 ± 0.44^b^	23.41 ± 0.17^b^	22.26 ± 0.34^c^	21.28 ± 0.04^e^
Butyric acid	1.53 ± 0.04^c^	3.01 ± 0.27^a^	3.00 ± 0.04^a^	2.01 ± 0.28^b^	1.46 ± 0.26^d^	0.47 ± 0.02^e^
Iso-butyric acid	0.70 ± 0.27^c^	1.69 ± 0.22^a^	1.66 ± 0.07^a^	0.84 ± 0.47^b^	0.29 ± 0.28^d^	ND
Propionic acid	1.34 ± 0.12^c^	2.15 ± 0.31^a^	2.13 ± 0.29^a^	1.52 ± 0.21^b^	1.36 ± 0.17^c^	0.50 ± 0.05^d^
Valeric acid	1.70 ± 0.13^b^	2.83 ± 0.28^a^	2.81 ± 0.46^a^	1.60 ± 0.09^b^	0.86 ± 0.03^c^	0.33 ± 0.13^d^
Iso-valeric acid	1.44 ± 0.05^b^	2.78 ± 0.13^a^	2.71 ± 0.02^a^	1.44 ± 0.07^b^	0.84 ± 0.22^c^	ND
Caproic acid	1.77 ± 0.01^d^	3.63 ± 0.18^a^	3.58 ± 0.02^b^	2.02 ± 0.04^c^	1.08 ± 0.19^e^	0.56 ± 0.20^f^

Data are mean ± SD values calculated from three replicate

Different letters (a-f) in each row indicate a significant difference between means (*p* < 0.05). ND; non detected

### Cytotoxic effect of *E. coli* KUB-36 metabolite and individual SCFA on Cancer Cells

Generally, MTT assay was used to determine the cytotoxicity, viability and proliferation of living cells. In this study, the concentration that inhibit 50% growth (IC_50_) of MCF10A normal breast cell, MCF7 breast cancer cell, HT-29 colon cancer cell and THP-1 leukaemia cancer cell was determined for 24, 48 and 72 h of incubation to compare the cytotoxicity effects of *E. coli* KUB-36 metabolites and individual SCFA that produced by *E. coli* KUB-36.

**Fig. 2:**
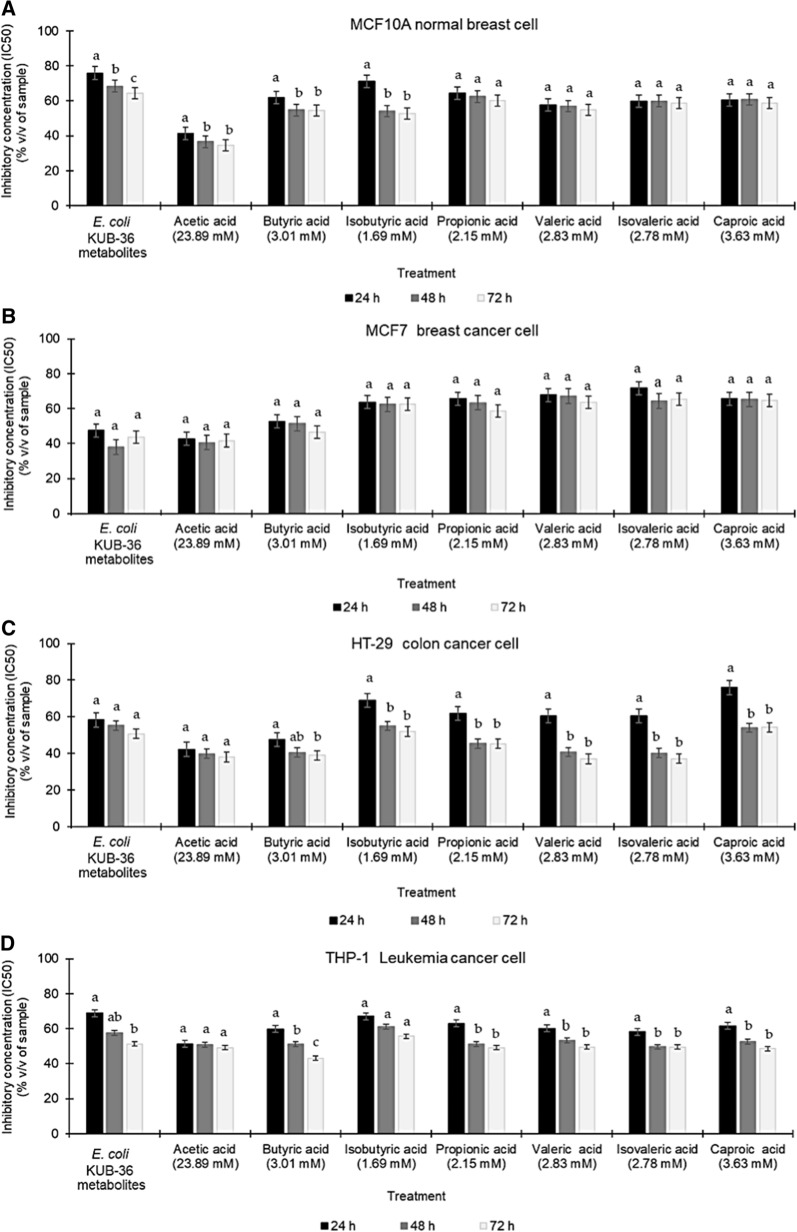
Cytotoxicity effects of *E. coli* KUB-36 metabolites and its individual SCFA on various cancer cells. **a** MCF10-A normal breast cell, **b** MCF7 breast cancer cell, **c** HT-29 colon cancer cell and **d** THP-1 leukaemia cancer cell. The initial concentration of individual SCFA for cytotoxicity analyses was prepared according to the highest concentration of SCFA (Acetic acid = 23.89 mM; Butyric acid = 3.01; Isobutyric acid = 1.69 mM; Propionic acid = 2.15 mM; Valeric acid = 2.83 mM; Isovaleric acid = 2.78 and Caproic acid = 3.63 mM) that present in *E. coli* KUB-36 metabolites at 8 h of incubation. Data are plotted as average values of IC_50_ ± standard error. Different lowercase (a–c) letters indicate significant difference (p < 0.05) between mean scores of each incubation in the column

For MCF10A normal breast cell, the IC_50_ value of the *E. coli* KUB-36 metabolites were 75.96%, 68.49% and 64.39% of the original concentration for 24, 48 and 72 h of incubation respectively, which were the lowest cytotoxicity activity as compared to all individual SCFA that were present in the *E. coli* KUB-36 metabolites, indicating that the *E. coli* KUB-36 metabolites has very limited cytotoxicity against the normal MCF10A breast cell. Nevertheless, acetic acid has exerted the highest cytotoxicity activity on MCF10A breast cell (Fig. 2a) as compared to all individual SCFA that present in the *E. coli* KUB-36 metabolites.

In comparison, MCF7 breast cancer cells have demonstrated a reverse trend of cytotoxicity activity, whereby the *E. coli* KUB-36 metabolites elicited more significant cytotoxicity as compared to other individual SCFA with the IC_50_ value of 48.84%, 37.99% and 43.79 of the original concentration detected at 24, 48 and 72 h of incubation respectively. Surprisingly, acetic acid also exerted a similar cytotoxicity effect on MCF7 breast cancer cell as shown in Fig. 2b.

For HT-29 colon cancer cell, the *E. coli* KUB-36 metabolites have exerted an IC_50_ value of 58.19%, 55.31% and 50.68% of the original concentration respectively for 24, 48 and 72 h of incubation. However, the acetic acid and butyric acid exerted better cytotoxicity activity as compared to *E. coli* KUB-36 metabolites. In addition, significant cytotoxicity activity was detected for 48 and 72 h of incubation for acetic acid, butyric, valeric and isovaleric acids as shown in Fig. 2c.

As for the THP-1 leukaemia cancer cell, the lowest cytotoxicity activities were detected for *E. coli* KUB-36 metabolites and the individual SCFA as compared to other cell lines, whereby an IC_50_ value of 68.90%, 57.54% and 51.98% of the original concentration were detected for 24, 48 and 72 h of incubation respectively for *E. coli* KUB-36 metabolites, suggesting that the *E. coli* KUB-36 metabolites and the individual SCFA that produced by *E. coli* KUB-36 have very little cytotoxicity against leukaemia cells as shown in Fig. 2d.

**Fig. 3:**
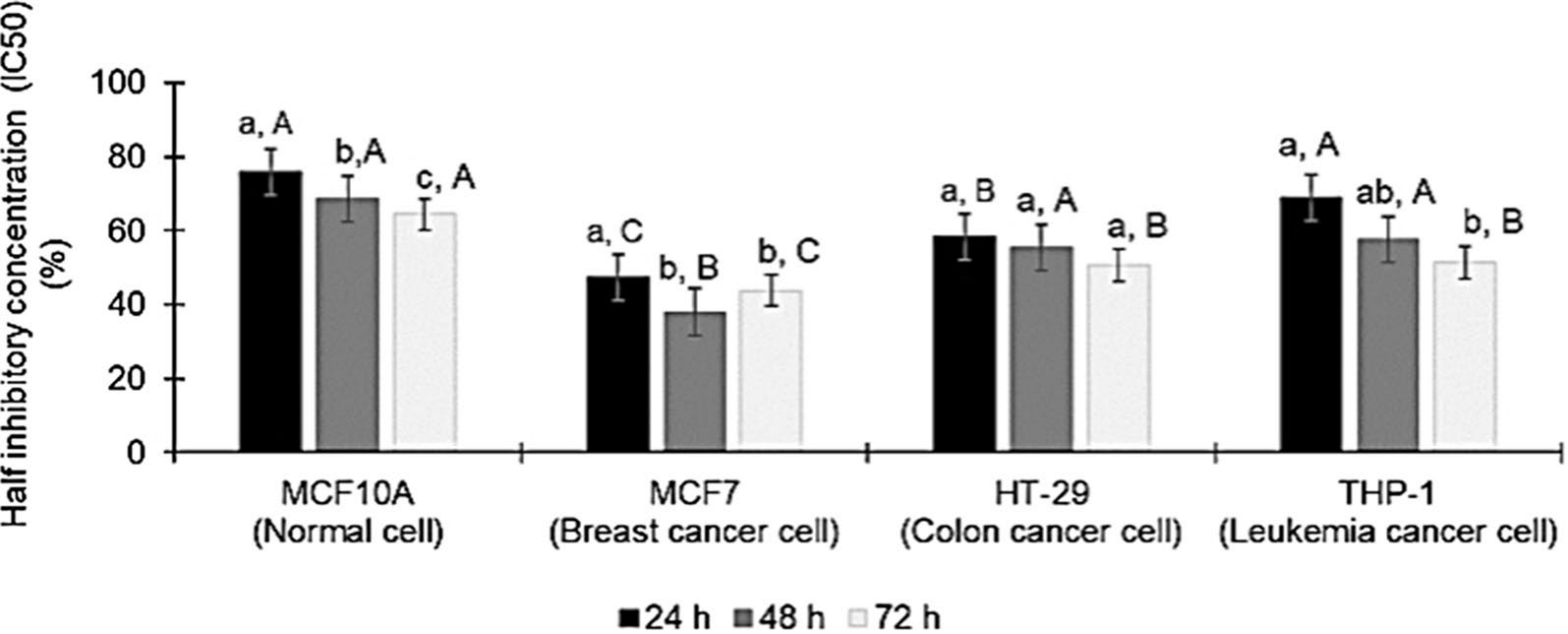
Cytotoxicity effects of *E. coli* KUB-36 metabolites on various cancer cells. Data are plotted as average values of IC_50_ ± standard error. Different lowercase (a-b) letters indicate (*p* < 0.05) significant difference between mean scores of each incubation time in the column. Different uppercase (A–C) letters indicate different (*p* < 0.05) between mean scores of each cancer cell type

Interestingly, the IC_50_ value of various cancer cell lines indicated that the *E. coli* KUB-36 metabolites treatment elicited more potent cytotoxicity effect on MCF7 breast cancer cell as compared to colon cancer cell and THP-1 leukaemia cancer cell. Moreover, Fig. 3 shows that the cytotoxicity effect of *E. coli* KUB-36 cell-free supernatant on colon cancer cell was not significantly different at 24, 48 and 72 h. In contrast, the cytotoxicity effect at 24 and 72 h was significantly different for THP-1 leukaemia cancer cells.

### Nitric oxide production and cytokine gene expression of LPS stimulated THP-1 macrophage cell

**Fig. 4:**
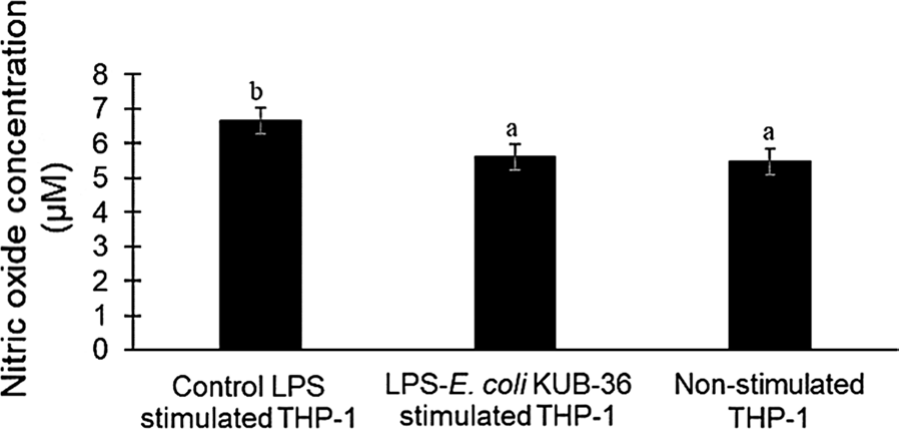
Nitric oxide production of LPS stimulated THP-1 macrophage cells. Nitric oxide production was determined after 6 h stimulation by 700 ng/mL control LPS and 700 ng/mL LPS of *E. coli* KUB-36. Data are plotted as average values of nitric oxide concentration ± standard error. Different lowercase letters (a–b) indicate significant differences (*p* < 0.05) between mean score of columns

**Fig. 5:**
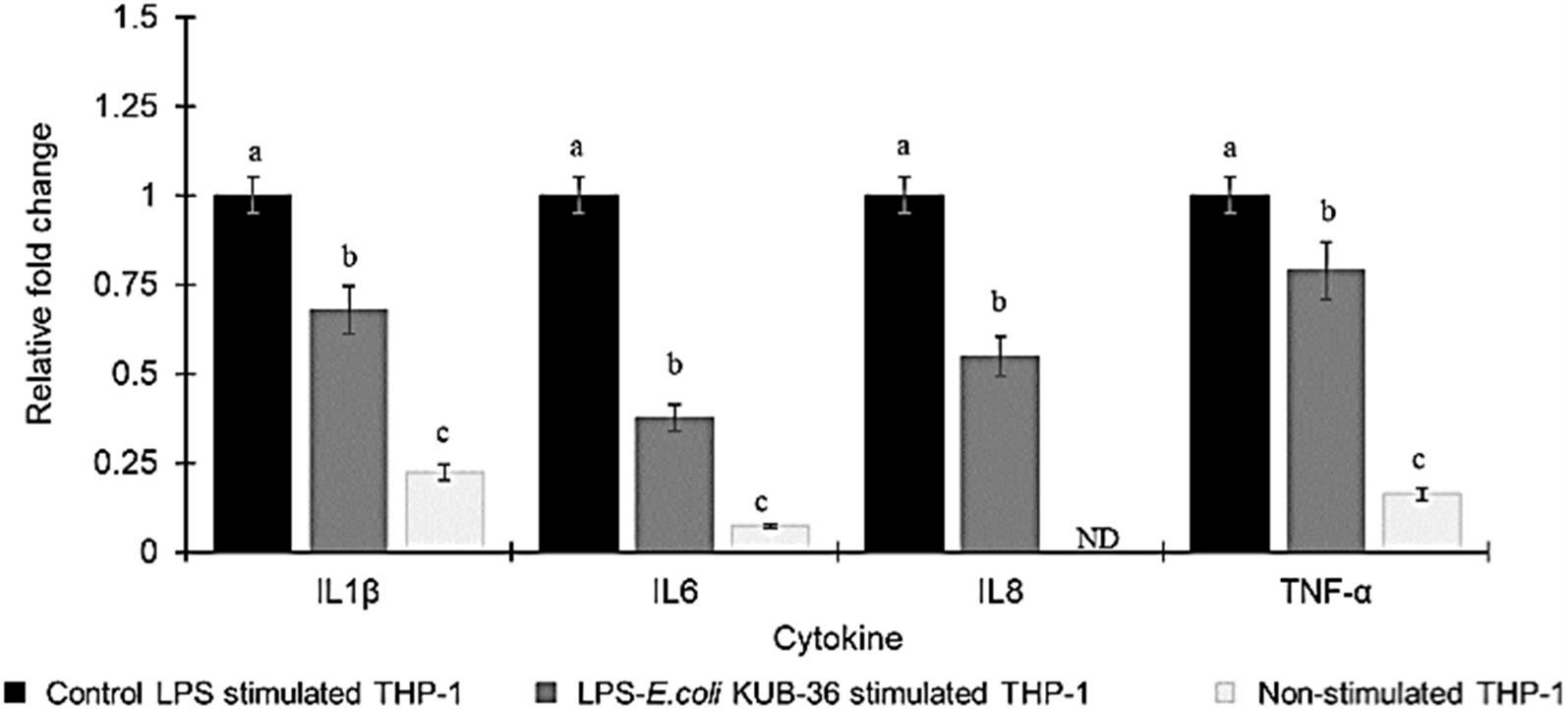
Inflammatory cytokine gene expressions of LPS stimulated THP-1 macrophage cells. The inflammatory cytokine gene expressions were determined for non-stimulated THP-1 macrophage cells and THP-1 macrophage cells stimulated by 700 ng/mL control LPS and 700 ng/mL LPS KUB-36 for r 6 h. Inflammatory cytokine gene expression was expressed relative to THP-1 stimulated by control LPS, which was calculated by 2^−ΔΔCt^ method. Data are plotted as average values of relative fold change ± standard error. Different lowercase letters (a–c) indicate (*p* < 0.05) significant differences (*p* < 0.05) between mean score from 6 replicates of each column

LPS is a major component of the outer membrane of Gram-negative bacteria that induces strong immune responses and plays a key role in inflammatory activities. In addition, nitric oxide (NO) is an important pro-inflammatory mediator produced by inducible NO synthase and cyclooxygenase-2 (COX-2). Moreover, the cytokine genes IL-8, IL-1β, IL-6, TNF-α and IL-10 are the regulators of pro- and anti-inflammatory immune responses. Therefore, the nitric oxide production and cytokine gene expression of THP-1 macrophage cells stimulated with control LPS and LPS of *E. coli* KUB-36 were investigated in this study as shown in Fig. 4. The results of NO production revealed that the highest NO production by THP-1 macrophage cells was stimulated by the control LPS as compared to the LPS of *E. coli* KUB-36, which was not significantly different from the non-stimulated THP-1 macrophage cells. This suggests that the LPS of *E. coli* KUB-36 did not induce any immune response and hence it did not have effect on inflammatory stimulation. The cytokine gene expressions of THP-1 macrophage cells stimulated by control LPS and LPS of *E. coli* KUB-36 were analysed by qPCR as shown in Fig. 5. The results showed that the expressions of cytokine IL-8, IL-1β, IL-6 and TNF-α were induced substantially by the control LPS in comparison to LPS of *E. coli* KUB-36.

### Effects of *E. coli* KUB-36 metabolites and individual SCFA on nitric oxide production and cytokine gene expression of LPS-stimulated THP-1 Macrophage cells

**Fig. 6:**
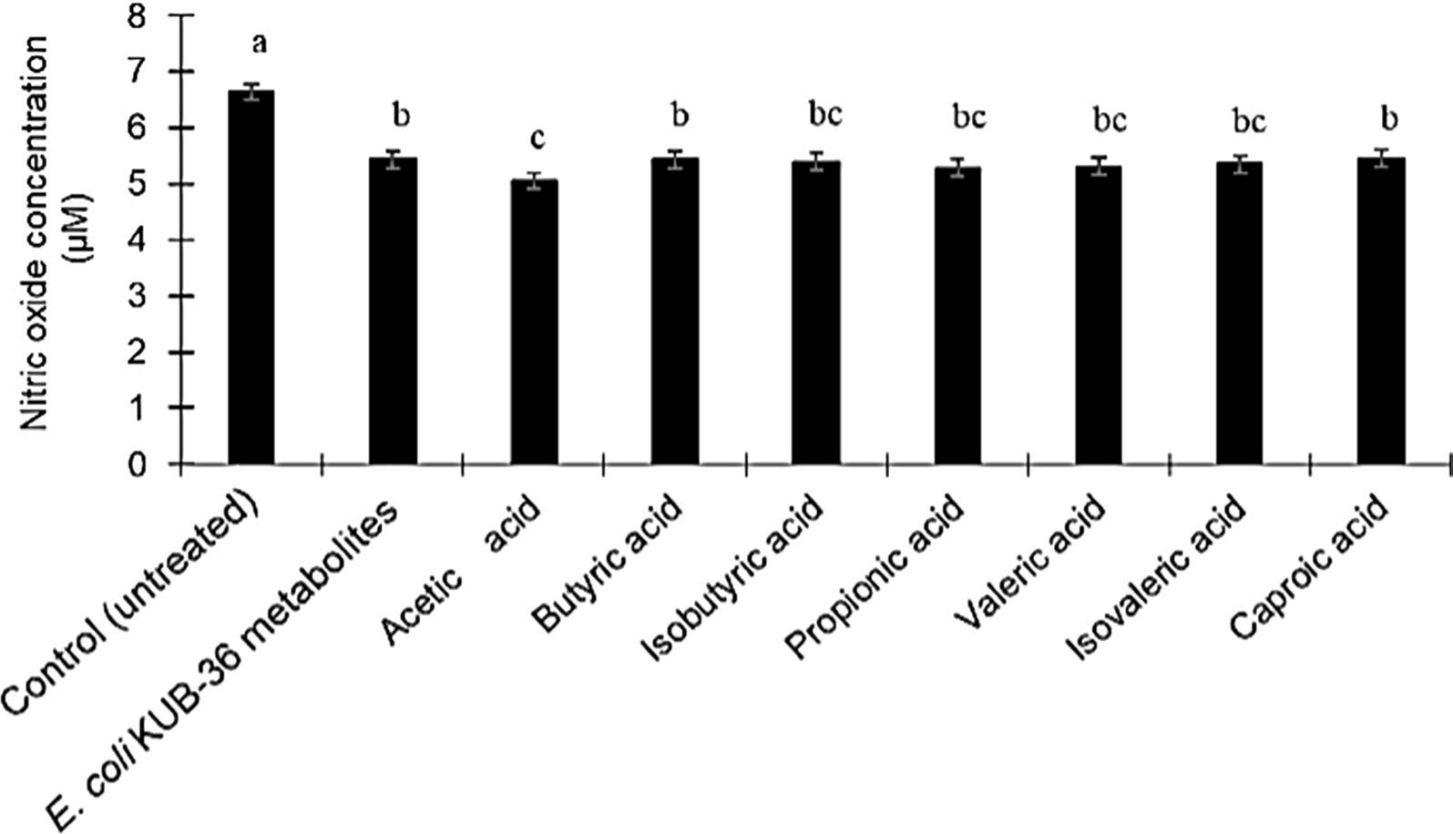
Effects of *E. coli* KUB-36 metabolites and its individual SCFA on nitric oxide production of LPS-stimulated THP-1 macrophage cells. IC_50_ values of *E. coli* KUB-36 metabolites and its individual SCFA were used in this experiment [*E. coli* metabolite; IC_50_ = 68.90%, Acetic acid; IC_50_ = 51.30% (12.26 mM); Butyric acid; IC_50_ = 59.88% (1.80 mM); Isobutyric acid; IC_50_ = 67.07% (1.13 mM); Propionic acid; IC_50_ = 63.63% (1.37 mM); Valeric acid; IC_50_ = 60.08% (1.70 mM); Isovaleric acid; IC_50_ = 58.27% (1.62 mM); Caproic acid; IC_50_ = 61.56% (2.23 mM)]. Data are plotted as average values of nitric oxide production ± standard error. Different lowercase letters (a-c) indicate significant differences (*p* < 0.05) between mean scores of columns

This study was conducted to investigate the inhibitory effect of *E. coli* KUB-36 metabolites and the individual SCFA on the production of NO by LPS-stimulated THP-1 macrophage cells. The level of NO production of LPS-stimulated THP-1 macrophage cells was determined by using a nitric oxide detection kit (iNtRON Biotechnology, Incheon, Korea). LPS-stimulation should up-regulate the NO production in THP-1 macrophage cells. However, Fig. 6 shows that the NO production of LPS-stimulated THP-1 macrophage cells was decreased after treatment with *E. coli* KUB-36 metabolites or individual SCFA. Generally, the NO production of *E. coli* KUB-36 metabolites treated of LPS-stimulated THP-1 macrophage cells was not significantly different from the NO production of individual SCFA treated of LPS-stimulated THP-1 macrophage cells, with the exception of acetic acid, where significant inhibitory effect was noted as compared to *E. coli* KUB-36 metabolite, butyric acid and caproic acid respectively.

**Fig. 7:**
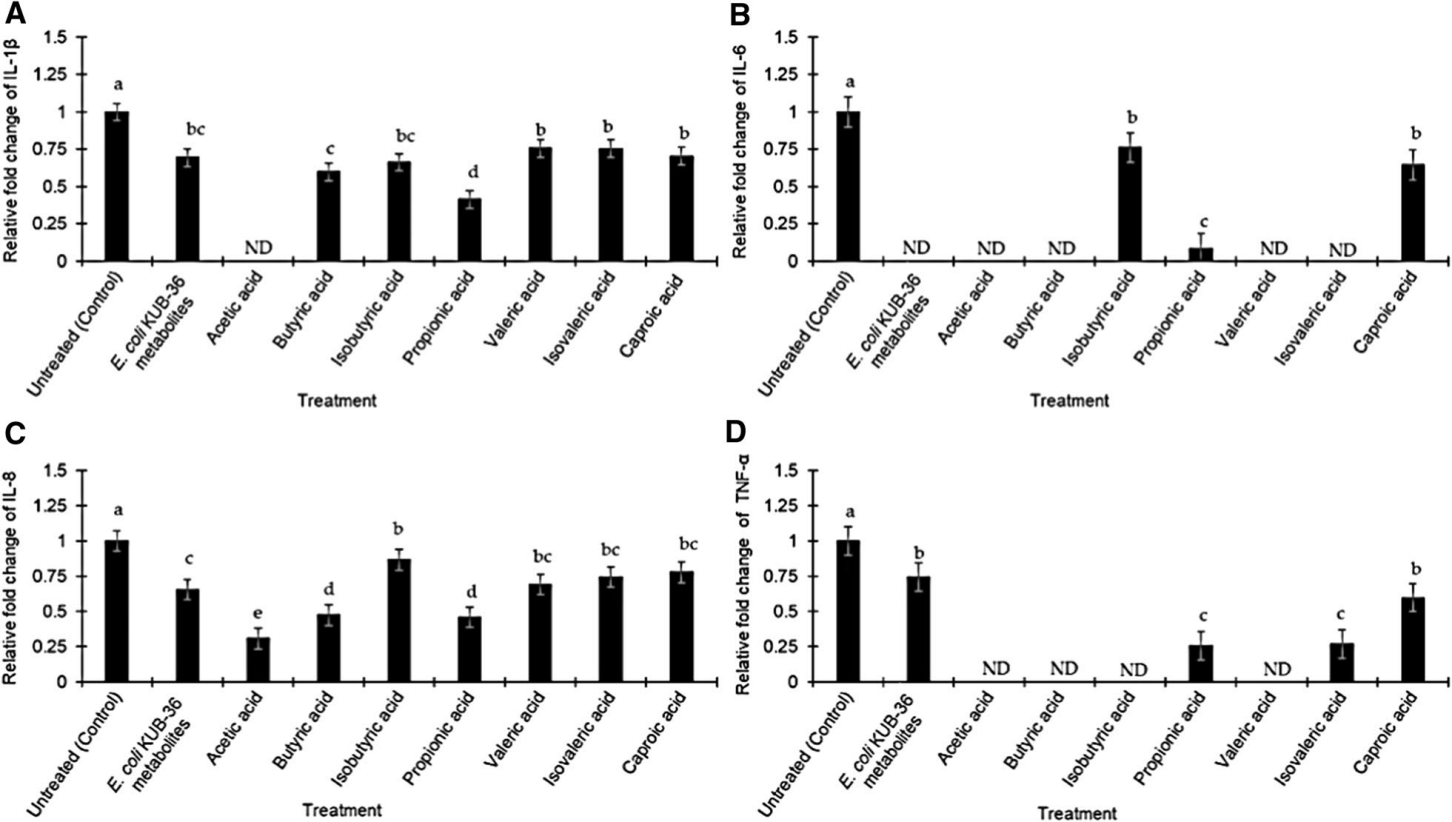
Effects of *E. coli* KUB-36 metabolites and its individual SCFA on inflammatory cytokine gene expressions of LPS stimulated THP-1 macrophage cells. **a** Inflammatory cytokine IL-1β expression. **b** Inflammatory cytokine IL-6 expression. **c** Inflammatory cytokine IL-8 expression and **d** inflammatory cytokine TNF-α expression. IC_50_ values of *E. coli* KUB-36 metabolites and its individual SCFA were used in this experiment [*E. coli* metabolite; IC_50_ = 68.90%, Acetic acid; IC_50_ = 51.30% (12.26 mM); Butyric acid; IC_50_ = 59.88% (1.80 mM); Isobutyric acid; IC_50_ = 67.07% (1.13 mM); Propionic acid; IC_50_ = 63.63% (1.37 mM); Valeric acid; IC_50_ = 60.08% (1.70 mM); Isovaleric acid; IC_50_ = 58.27% (1.62 mM); Caproic acid; IC_50_ = 61.56% (2.23 mM)]. THP-1 macrophages cells were treated with *E. coli* KUB-36 metabolites and its individual SCFA for 3 h before exposure to LPS for 6 h. Inflammatory cytokine gene expression was expressed relative to THP-1 macrophages cells stimulated by control LPS, which was calculated by 2^−ΔΔCt^ method. Data are plotted as average values of relative fold change ± standard error. Different lowercase superscript letters (a–d) shown in the column indicate significant differences (*p* < 0.05) between mean scores of each column from 6 replicates

Subsequently, the inhibitory effect of *E. coli* KUB-36 metabolites and individual SCFA were investigated for the expression of inflammatory cytokines by the LPS-stimulated THP-1 macrophage cells. Relative fold changes of 4 important inflammation-related gene expressions (cytokine IL-1β, IL-6, IL-8 and TNF-α) that attributed to the LPS stimulation was used as a control for the inflammatory condition. Therefore, the level of cytokine gene expression that was above or below the control level was designated as inductive or suppressive inflammatory responses, respectively. Figure 7 shows the effect of *E. coli* KUB-36 metabolites and individual SCFA on cytokine gene (IL-1β, IL-6, IL-8 and TNF-α) expressions of LPS-stimulated THP-1 macrophage cells. Interestingly, *E. coli* KUB-36 metabolites and its individual SCFA resulted in more than 80% suppression of the inflammatory cytokine IL-1β expression as compared to the control. However, the individual SCFA exerted better suppression on inflammatory cytokine IL-1β expression as compared to the *E. coli* KUB-36 metabolites, except for the iso-valeric acid, which was not significantly different from *E. coli* KUB-36 metabolites. Surprisingly, acetic acid completely suppressed the inflammatory cytokine IL-1β expression (Fig. 7a), which have the same suppression profile as the inflammatory cytokine IL-6 expression. The results showed that *E. coli* KUB-36 metabolites and individual SCFA that produced by *E. coli* KUB-36 metabolites have high suppression on the inflammatory cytokine IL-6 expression as compared to the control. Complete suppression of inflammatory cytokine IL-6 expression was noted for *E. coli* KUB-36 metabolites, acetic acid, butyric acid, valeric acid and isovaleric acid (Fig. 7b). In comparison to the inflammatory cytokine IL-8 expression, *E. coli* KUB-36 metabolites and individual SCFA have lower suppression on the inflammatory cytokine expression than IL-1β and IL-6. Moreover, the individual SCFA still have better suppression on inflammatory cytokine IL-8 expression as compared to the *E. coli* KUB-36 metabolites, especially acetic acid which showed the highest suppression of inflammatory cytokine IL-8 expression (Fig. 7c).

As for the inflammatory cytokine TNF-α expression, the results obtained in this study showed that *E. coli* KUB-36 metabolites could suppress inflammatory cytokine TNF-α expression, but with lower suppression activity than the inflammatory cytokine IL-1β and IL-6 expression. Nevertheless, the individual SCFA, especially, acetic, butyric, iso-butyric and valeric acids elicited induced complete suppression on the inflammatory cytokine TNF-α expression (Fig. 7d).

Furthermore, the anti-inflammatory cytokine IL-10, which is the most important anti- inflammatory gene (normally expressed by macrophage cells) was also determined in this study. The results showed that the control did not express the IL-10 gene. Therefore, relative quantification or fold change could not be determined. Nonetheless, the treated sample showed indication of IL-10 expression (data not shown), hence, the results of IL-10 gene expression was normalized to the housekeeping genes (GAPDH and ACTP) and expressed as ΔCt value in present study. The ΔCt value of *E. coli* KUB-36 metabolites, iso-butyric acid, propionic acid and caproic acid were 5.10, 5.22, 2.26 and 3.56 respectively (data not shown), indicating that *E. coli* KUB-36 metabolites, iso-butyric acid, propionic acid and caproic acid induced the expression of anti-inflammatory cytokine IL-10. However, acetic acid did not induce anti-inflammatory cytokine IL-10 expression.

## Discussion

The human gut microbiota, especially the probiotic bacteria play an important role in gut homeostasis and have an impact on host metabolism and health. Among probiotic metabolites, SCFA is one of the postbiotic component that contributes greatly to maintain the GI tissue integrity and may positively affect the body’s immune response [38]. Therefore, we selected the highest butyric acid producing bacteria, which was designated as *E. coli* KUB-36 for toxin gene detection, SCFA production profiles, anti-cancer and anti-inflammatory activities induced by its metabolites. The anti-cancer and anti-inflammatory effects of individual SCFA that present in the *E. coli* KUB-36 metabolites were further verified in this study. In current study, *E. coli* KUB-36 were grown in vitro by using a synthetic medium of Wilkins Chalgren under anaerobic condition to produce SCFA and cell growth. The in vitro cultured condition was different from the *in*-*vivo* gut environment, whereby innate immune responses, anti-microbial peptides, oxygen barrier, secretory IgA, epithelial microvilli, epithelial tight junctions, epithelium metabolism, mucus barrier and quorum sensing were absent.

*E. coli* is a type of bacteria that normally lives in the intestine of human [39]. Most *E. coli* are harmless and help in the health maintenance of the digestive tract [40]. In this study, we observed that *E. coli* KUB-36 was rapidly grown in the Wilkina Chalgren medium and the greatest amounts of SCFA was produced after 8 h of incubation. In comparison, *E. coli* KUB-36 produced mainly acetic acid as a key product and minor amounts of butyric, isobutyric, propionic, valeric, isovaleric and caproic acids. The SCFA profile of *E. coli* KUB-36 was similar to probiotic *E. coli* Nissle 1917, whereby acetic acid and formic acid were produced mainly as compared to the minor amounts of propionic and butyric acids [41]. Acetic acid can be utilised as an energy source by the gut bacteria. Therefore, *E. coli* KUB-36 might be beneficial since it induced anti-inflammatory response while producing energy for other gut microbiome. The beneficial bacteria are mostly related to probiotic lactic acid bacteria and reports on SCFA are mostly based on whole metabolites produced by bacteria. Hence, in present study, we report the occurrence of beneficial SCFA-producing *E. coli* and the probiotic effects of individual SCFA that present in *E. coli* metabolites, which have not been reported elsewhere.

Although *E. coli* strains in the human intestine are harmless, the outer membrane of *E. coli* contains the potent immunostimulatory molecule lipopolysaccharide, which is known as endotoxin. Additionally, some *E. coli* strains harbour enterotoxin genes that are responsible for diarrhoea incidents [42, 43]. Therefore, the presence of both endotoxin and enterotoxin genes in *E. coli* KUB-36 were verified in this study. Endotoxin that is known as LPS will be released upon destruction of the bacterial cell wall [44]. Endotoxins consist of: a) lipid A which is responsible for the toxicity of Gram-negative bacteria that can be recognized by the host innate immune system, b) a core polysaccharide that links the lipid A to the O-antigen, the outermost part of the LPS molecule expressed on the bacteria cell wall, which is the major antigen targeted by the host antibody responses.

The lipid A biosynthetic pathway in *E. coli* comprises nine constitutive enzymes, which are LpxA, LpxB, LpxC, LpxD, LpxH, LpxK, LpxL, LpxM and WaaA [45]. Some *E. coli* may not harbour some of the genes encoding the lipid A biosynthetic enzymes*.* However, the genes encoding the first four enzymes *(*LpxA, LpxC, LpxD and LpxB*)* are often present in *E. coli* [46]. In this study, we could amplify the first four genes encoding lipid A: *lpxA, lpxB, lpxC* and *lpxD*, which have been reported for *E. coli.* Lipid A of *E. coli* induces an inflammatory reaction in host cells. However, structurally different lipid A can result in weak inflammatory responses in the host and hence, it can be completely blocked by any pro-inflammatory reactions via binding to the corresponding host receptors [47]. The biosynthesis of the core polysaccharide is encoded by *waaF* and *waaC* genes. Nevertheless, *wzy* and *wzz* genes are the important genes that responsible for O-antigen biosynthesis, whereby the *wzy* gene is involved in the polymerization of O-antigen subunits, while the *wzz* gene functions as a molecular ruler to determine the O-antigen chain length that ligates to the terminal sugar residues of the core-lipid A in a reaction mediated by *waaL*. For comparison, only the *waaF* and *wzy* genes encoding the core polysaccharide and O-antigen were amplified from *E. coli* KUB-36 in this study. The *E. coli* KUB-36 did not habour both *waaL* and *wzz* genes, which mediates the ligation of O-antigen onto lipid A-core and the regulation of the length of polysaccharide O-antigen [48] respectively. Similar results were also reported for commensal *E. coli* K-12 [49]. Therefore, *E. coli* KUB-36 most likely possessed a defective LPS biosynthetic pathway without the presence of O-antigen, attributing to the absent of the *wzz* and *waaL* genes. Likewise, both heat-labile and heat-stable enterotoxin genes were also absent from *E. coli* KUB-36. Similarly, both probiotic *E. coli* Nissle 1917 and non-pathogenic *E. coli* K-12 did not harbour heat-labile and heat-stable enterotoxins gene [6, 47, 50–53]. Therefore, the results of this study implied that *E. coli* KUB-36 is most likely a safe commensal bacterium.

Nowadays, bacteria are proposed to be used as an effective biotherapeutic agent in disease treatments, especially the gut microbiota. Cancer is reported to be a leading cause of death globally and is responsible for an estimated 9.6 million deaths in 2018 [9]. The increasing incidents of drug resistance in cancer treatment has urged a drastical and urgent attempt to search for a new drug for cancer treatments. Gut bacteria that could produce SCFA metabolites have been demonstrated to exert anti-cancer effects [11–16]. Moreover, SCFA are generally the fermentation product of gut microbiota. Importantly, SCFA have a role in gut and immune homeostasis, attributing to their potential anticancer properties, lipid metabolism, anti-inflammatory and other immune effects, including atopic diseases [4]. In this study, *E. coli* KUB-36 metabolites was demonstrated to have cytotoxicity effect on breast cancer cell, colon cancer cell and leukaemia cancer cell at 24, 48 and 72 h of incubation, whereby the highest cytotoxicity activity was detected for MCF7 breast cancer cell at 48 and 72 h of incubation. Furthermore, the individual SCFA also exhibited similar cytotoxicity effect as *E. coli* KUB-36 metabolites in a time and dose dependent manner. Previous studies have reported that 2–5 mM of butyric acid could reduce the cell viability of colon cancer cell [54], breast cancer cell [55], and leukaemia cancer cell [56] respectively. Acetic and propionic acids at a concentration between 2–10 mM and valeric acid at a concentration of 5 mM could reduce the cell viability of colon cancer cell [54, 57]. In addition, caproic acid at a concentration between 0.6–44 mM exhibited anti-cancer activity on colon, skin and breast cancer cells [58]. Thus, the cytotoxicity activities of *E. coli* KUB-36 metabolite were most likely to be attributed to the extracellular SCFA produced by *E. coli* KUB-36. In the human host, the SCFA produced by the microbiota in the cecum and colon can be found in the hepatic, portal and peripheral blood circulation. However, most of the propionic and butyric acid from portal circulation are degraded in the liver to prevent high SCFA concentration in the blood circulation. Acetate is the only SCFA distributed throughout the circulation [3]. Therefore, the observed in vitro effects of cytotoxicity activity on breast cancer cells would differ considerably as compared to in vivo condition.

Proinflammatory cytokines are commonly induced by the LPS cell wall component of Gram-negative bacteria. Therefore, in this study we have made an attempt to investigate the structure of LPS of *E. coli* KUB-36, which could be involved in the immune response by inducing cytokines expression (IL-1β, IL-6, IL-8 and TNF-α) and NO production. NO production of stimulated THP-1 cell with LPS of *E. coli* KUB-36 was not significantly different from the non-simulated THP-1 cell, which could be due to the absence of some core-polysaccharide and O-antigen in the LPS of *E. coli* KUB-36. Likewise, the LPS of *E. coli* nissle 1917 was shown to have a very short O-antigen polysaccharide side chain, whereby a stop-codon in the open reading frame (ORF) encoded the O-antigen polymerase [52] was detected in *E. coli* nissle. However, the LPS of *E. coli* KUB-36 induced IL-1β, IL-6, IL-8 and TNF-α proinflammatory cytokines, despite having lower effect than the LPS of pathogenic bacteria since a defective lipid A was found on *E. coli* KUB-36 membrane. Thus, the immune response of the LPS was mainly induced by the lipid A moiety [23]. Similarly, the LPS from outer membrane of *E. coli* Nissle 1917 has been reported to elicit pro-inflammatory cytokine IL-1β, IL-6, TNF-α and VEGF production [4, 59]. However, *E. coli* Nissle 1917 metabolites have been shown to reduce pro-inflammatory cytokine, but nevertheless induced anti-inflammatory cytokine [6], whereby the LPS of *E. coli* Nissle 1917 strain exhibited immunomodulating properties without showing immunotoxin effects [52].

Inflammation may be a trigger factor in various cancer incidents. In general, the longer persistence of inflammation will impose a higher risk to cancer incidents, whereby inflammatory mediators include cytokines, chemokines and free radicals that will lead to increased cell proliferation, mutagenesis oncogene activation and angiogenesis which promote cancer cell development. However, SCFA have been reported to exert anti-inflammatory effect, such as the reduction of IL-8 production during airway inflammation via the activation of free fatty acid receptors 2 and 3 (FFA) of macrophages [60]. Furthermore, butyric and propionic acid have been shown to decrease LPS-induced TNFα and nitric oxide synthase (NOS) expression in monocytes [61]. Butyric acid upregulates IL-10 production and suppresses the production of pro-inflammatory molecules of IL-12, TNFα, IL-1β and NO by inhibiting NF-κB activity [12, 16, 62]. Butyric acid also can decrease inducible NOS (iNOS), TNFα, MCP-1 and IL-6 production by activation of FFA3 receptor [63] of macrophage cell. In addition, butyric and propionic acids have been demonstrated to suppress TNFα production and NF-κB activity, while promoting the production of anti-inflammatory cytokine IL-10 in LPS-activated mononuclear cells by inhibiting the histone deacetylases (HDACs) [14, 64, 65], whereas propionic acid was reported to inhibit NO production by NF-κB activation of macrophages [16] and acetic acid was reported to inhibit LPS-induced TNFα secretion from human mononuclear cells by activating FFA receptor pathways [16]. Therefore, we have made an investigation on the NO production and anti-inflammatory effect of *E. coli* KUB-36 metabolite and individual SCFA.

In this study, *E. coli* KUB-36 metabolite and individual SCFA reduced the production of NO and proinflammatory cytokines IL-1β, IL-6, IL-8 and TNF-α, inferring that *E. coli* KUB-36 metabolites and individual SCFA could affect inflammatory responses in LPS stimulated THP-1 macrophage cells. Moreover, the results obtained in this study implied that *E. coli* KUB-36 metabolites has similar effect to *E. coli* nissle 1917 metabolites, which could inhibit the production of cytokine IL-2, TNF-α, and IFN-γ, while promoting the production of anti-inflammatory cytokine IL-10 [6]. Nevertheless, the results of this study revealed that *E. coli* KUB-36 metabolite induced the gene expression of anti-inflammatory cytokine IL-10, despite individual acetic, butyric and propionic acid did not induce the anti-inflammatory cytokine IL-10 gene expression, suggesting that *E. coli* KUB-36 metabolites might contain other bioactive compounds that could induce the anti-inflammatory cytokine IL-10 expression.

## Conclusion

In conclusion, this study demonstrated that the *E. coli* strains isolated from healthy human faeces produced different SCFA profile which could be considered as a safe commensal bacterium since they did not possess any toxin gene. The extracellular metabolites produced by the selected *E. coli* KUB- 36 strain exhibited lower cytotoxicity activity on normal MCF10A breast cell and THP-1 leukaemia cell but demonstrated higher cytotoxicity activity on both HT-29 colon cancer and MCF7 breast cancer cell. Furthermore, *E. coli* KUB-36 metabolites displayed anti-inflammatory effects on LPS-induced macrophage cells, since it suppressed well in the production of inflammatory cytokine IL-6, IL-8, IL-1β and TNF-α. In comparison, *E. coli* KUB-36 metabolites that contained mainly acetic acid conferred highly positive effects on anti-inflammatory activity of macrophage cells by inducing the production of IL-6, IL-1β and TNF-α. The overall results of this study have provided strong evidence to support *E. coli* KUB-36 as a potential probiotic bacterium that could be used as a bioagent for inflammatory prevention and chemopreventive agent for cancer development and treatment.

## Methods

### Isolation of gut bacteria and maintenance

Ethics approval for this study was obtained within the framework of the study code KUREC-HS61/003 (2/April/2018) given by the Kasetsart University Research Ethics Committee. The faecal sample was collected from three healthy volunteers aged between 25 and 60 years that was selected based on the following criteria: normal body mass index, did not have diseases, non-smoking, non-alcohol drinking, practice routine exercise and annual routine medical check-up. The faeces were aseptically transferred into a sterile tube containing Wilkins Chalgren anaerobic broth (Oxford, Basingstoke, UK) prior to flushing with CO_2_. The faeces samples were subjected to tenfold serial dilutions and mixed by vertexing for 3 min. Total viable count was performed by using Wilkins Chalgren anaerobic agar that was incubated at 37 °C under an anaerobic system (Bastron anaerobic chambers, Sheldon Co., Ltd., USA) fed with 5% CO_2_, 5% H_2_ and 90% N_2_ for 48 h. Each colony was isolated by re-streaking on Wilkins Chalgren anaerobic agar for 2–3 times. The isolates were then maintained by using 50% (v/v) glycerol and kept at − 20 °C until further use.

### Bacterial culture and metabolites preparation

Bacteria was revived by inoculation into Wilkins Chalgren anaerobic broth at 1% (v/v) and incubated at 37 °C for 24 h under anaerobic condition. The cultured supernatant of bacteria was collected and designated as metabolites after centrifugation at 2044×*g*, 4 °C for 15 min.

### Determination of short chain fatty acid profile

Gas chromatography (GC) is most used for SCFA analysis due to compatibility with the chemical properties of SCFA, such as volatility. Flame ionization detection (FID) is commonly used to detect the SCFA due to its inexpensive cost for operation, as well as its ability to detect a wide range of organic compounds. Therefore, GC was used for the determination of SCFA profile in this study. The SCFA profile of overnight cultured supernatant was analyzed by using GC (GC, Agilent 6890; Agilent Technologies, Palo Alto, CA, USA). The composition of acetic, propionic, iso-butyric, butyric, iso-valeric, valeric and caproic acids were determined for SCFA of cultured supernatant. To determine the SCFA concentration, 0.5 mL of 4-methyl-n-valeric acid (Sigma, Missouri, USA) was added to 0.5 mL of each cultured supernatant as an internal standard before being analysed by GC. The SCFA analysis was performed using a fused-silica capillary column (30 m × 0.25 µm × 0.25 µm) (Agilent 6890; Agilent Technologies, Palo Alto, California, USA) in a GC that equipped with FID detector. The flow rate of nitrogen as the carrier gas was 1.0 mL/ min. The running conditions for SCFA analyses were maintained at 160 °C with FID detector at 250 °C and injector temperature at 230 °C. The GC analyses for SCFA were conducted in triplicate for each metabolite.

### 16S rRNA sequence analysis

The selected bacteria were identified by performing a full length 16S rRNA sequencing using universal primer sequence of 27F and 1492R (Ward Medic Co., Ltd., Bangkok, Thailand). The full length of 16S rRNA sequences were aligned with the consensus sequences deposited at GenBank of NCBI and sequence alignment data were analysed by using BLAST software of the NCBI.

### DNA extraction

Genomic DNA of selected bacteria was prepared using a Genomic DNA extraction mini kit according to the instruction of manufacturer (Yeastern Biotech Co., Ltd*,* Taipei, Taiwan). Briefly, bacteria cells were cultured at 37 °C for 24 h under anaerobic condition before centrifuging at 14,000×*g* at room temperature for 1 min. The supernatant was then discarded and the bacterial cell pellet was resuspended with 50 μL N1 Buffer, followed by adding 300 μL of Lysis Buffer and incubated at 60 °C for 10 min until clear lysate was observed. To remove protein, 400 μL Protein Removal Buffer was then added to the lysate mixture prior to centrifugation at 14,000×*g* for 1 min, followed by washing the extracted DNA pellet twice with 400 μL of Washing Buffer. Wash solutions were removed by centrifugation at 14,000×*g* for 30 s and the DNA pellet was then dried by centrifugation at 14,000×*g* for 3 min. Purified DNA pellet was eluted with 90 μL of preheated elution buffer (75 °C) for 3 min, followed by centrifugation at 14,000×*g* for 2 min. The purified DNA extract was kept at − 20 °C until used.

### Toxin gene detection

The presence of LPS endotoxin genes (*lpxA, lpxB, lpxC, lpxD, waaC, waaF, waaL, waaQ, wzy* and *wzz*) and exotoxin genes (*etlB*, heat-labile; *Stb*, heat-stable) were verified for the selected *E. coli* strains using the primers listed in Table 1. The PCR condition for the amplification of the toxin genes were carried out as follows: DNA was denatured for 2 min at 95 °C and amplified for 30 cycles (30 s at 95 °C, 30 s at 48–63 °C, 1 min at 72 °C for denaturation, annealing and extension phases, respectively), followed by additional period of 10 min extension at 72 °C. PCR products were separated by electrophoresis using 1% (*w/v*) agarose gel and stained with 0.5% (*v/v*) nucleic acids stain. The amplified bands were visualized and recorded under UV illumination.

### Cell culture and maintenance

Colorectal cancer cells HT-29 were provided by the Tissue Culture Laboratory of UPM, whereas human breast cancer cells MCF-7, human normal breast cell MCF-10A (reference of normal glandular epithelium) and THP-1 leukaemia cancer cell were purchased from American Type Culture Collection (ATCC). Human normal breast cell MCF-10A was cultured in Dulbecco’s Modified Eagle Medium (DMEM) (Invitrogen, Carlsbad, CA, USA) supplemented with 0.01 mg/ml insulin and 10% fetal bovine serum (FBS), Human breast cancer cells MCF-7, cultured in DMEM supplemented with 20 ng/ml EFG, 0.5 µg/ml hydrocortisol, 5% horse serum, and 10 µg/ml insulin. Colorectal cancer cells HT-29 was cultured in RPMI (Gibco, USA) supplemented with 10% (v/v) FBS, 100 UI/ml of penicillin-streptomycin. All cells were maintained at 37 °C under 5% CO_2_ atmosphere.

### Cytotoxic effect of selected *E. coli* KUB-36 metabolites and individual SCFA

Normal and cancer cells were seeded onto 96-well microplates at 1 × 10^5^ cells/mL and incubated at 37 °C in a 5% CO_2_ incubator for cytotoxicity assay. The initial concentration of individual SCFA for cytotoxicity analyses was prepared according to the highest concentration of SCFA (Acetic acid = 23.89 mM; Butyric acid = 3.01; Isobutyric acid = 1.69 mM; Propionic acid = 2.15 mM; Valeric acid = 2.83 mM; Isovaleric acid = 2.78 and Caproic acid = 3.63 mM) that present in *E. coli* KUB-36 metabolites at 8 h of incubation (Table 2). For the determination of IC_50_ value, two-fold dilution was conducted for *E. coli* KUB-36 metabolite and individual SCFA by using growth medium to achieve the final concentrations of 1.56–100% (v/v). At the respective incubation time of 24 h, 48 h and 72 h, a volume of 20 μL of 3-(4,5-dimethylthiazol-2-yl)-2,5-diphenyl tetrazolium bromide (MTT) solution (Sigma, MO, USA) (5 mg/mL in PBS) was added to each well and incubated for 4 h in dark condition after removing the treatment mixture from each well. Then, 100 μL of dimethylsulfoxide (DMSO) (Fisher Scientific, Loughborough, UK) were added to each well and mix thoroughly by pipetting 10–20 times to dissolve the blue formazan crystals. The absorbance of the formazan dye was quantified by a Quant ELISA reader (Biotek EL340, Vermont, USA) at 570 nm with a reference wavelength of 630 nm. The experiment was repeated three times with triplicate samples. The following equation was used to calculate the percentage of cell viability:

$$\frac{{({\text{A}}_{{{\text{sample }}}} - {\text{ A}}_{{{\text{blank }}}} )}}{{({\text{A}}_{{{\text{control }}}} - {\text{A}}_{{({\text{blank}})}} )}} \times 100$$

A_sample_ represents the absorbance of cells treated with metabolites or individual SCFA; A_blank_ represents absorbance of the growth media and A_control_ represents absorbance of untreated cells. The concentration for 50% of growth (IC_50_) was determined by plotting the percentage of cell viability versus the concentration of metabolites and individual SCFA, respectively.

### Extraction of *E. coli* KUB-36 lipopolysaccharide

LPS of *E. coli* KUB-36 was extracted by using LPS extraction kit (100 Rxn, iNtRON Biotechnology, Incheon, Korea) according to the instruction of manufacturer. Briefly, 2–5 ml of *E. coli* KUB-36 cells were collected by centrifugation at 14,000×*g* for 10 min at room temperature, followed by adding 1 ml of lysis buffer to lysis the bacterial cell by vertexing vigorously. After the addition of 200 µl of chloroform, the lysis mixture was centrifuged at 14,000×*g* for 10 min at 4 °C prior to the collection of 400 µl of upper aqueous layer. A volume of 800 µl of purification buffer was then added to the 400 µl of upper aqueous layer before incubation at − 20 °C for 10 min. The extracted LPS was collected by centrifugation at 14,00×*g* for 15 min at 4 °C, followed by washing twice with 1 ml of 70% (v/v) ethanol. The washed LPS pellet was collected by centrifugation at 14,000×*g* for 3 min at 4 °C and the upper layer was discarded. The remaining LPS pellet was the allowed to dry at room temperature.

### Nitric oxide production

The NO concentration was determined by measuring the amount of nitrite in the cell culture supernatant using a Nitric Oxide Detection Kit (iNtRON Biotechnology, Incheon, Korea). A 100 µL aliquot of the cell culture supernatant was mixed with 100 µL of Griess reagent and the mixture was incubated for 10 min at room temperature. The absorbance was measured at 540 nm using a Quant ELISA reader (Biotek EL340, Vermont, USA).

### THP-1 cell differentiation

The human monocytic leukaemia cell line THP-1 (American Type Culture Collection, Rockville, MD) was grown in RPMI 1640 culture medium (Sigma, Neustadt, Germany) supplemented with 10% (*v/v*) FBS (Invitrogen, UK) and 1% (*v/v*) penicillin/streptomycin (P/S) (Invitrogen) at 37 °C in 5% CO_2_ humidified incubator. Cells were sub-cultured at 72 h intervals. The macrophage-like cells were obtained by treating 1 mL of 10^6^ THP-1 monocytes/well with 100 ng/mL phorbol 12-myristate 13-acetate (PMA; Sigma, Neustadt, Germany) for 48 h in a 12-wells cell culture plate (Greiner, Frankenhauser, Germany). Differentiated and plastic-adherent macrophage-like cells were then washed twice with RPMI culture medium and rested for another 24 h with culture medium to obtain the resting state of macrophages.

### Inflammation gene expression of THP-1 Macrophage

THP-1 macrophages were pre-incubated with culture medium mixed with IC_50_ concentrations of *E. coli* KUB-36 metabolite and individual SCFA (*E. coli* KUB-36 metabolite; IC_50_ = 68.90%, Acetic acid; IC_50_ = 51.30% (12.26 mM); Butyric acid; IC_50_ = 59.88% (1.80 mM); Isobutyric acid; IC_50_ = 67.07% (1.13 mM); Propionic acid; IC_50_ = 63.63% (1.37 mM); Valeric acid; IC_50_ = 60.08% (1.70 mM); Isovaleric acid; IC_50_ = 58.27% (1.62 mM); Caproic acid; IC_50_ = 61.56% (2.23 mM) in a 12-wells cell culture plate for 3 h prior to the additional incubation of 6 h with 700 ng/mL LPS. THP-1 macrophages treated with culture medium containing PBS was employed as the control for this experiment. The expression of inflammatory and anti-inflammatory genes (Table 3) was determined by using qPCR. The experiment was repeated twice with triplicate samples.

**Table 3:** Primer sequences of RT-qPCR used for the analyses of inflammatory and anti-inflammatory gene expressions of LPS-stimulated THP-1 macrophage cells

	Specific gene	Primer
Cytokines gene	*IL-1β*	F-GTGGCAATGAGGATGACTTGTTC R-TAGTGGTGGTCGGAGATTCGTA
	*IL-6*	F-AGCCACTCACCTCTTCAGAAC R-GCCTCTTTGCTGCTTTCACAC
	*IL-8*	F-CTGATTTCTGCAGCTCTGTG R-GGGTGGAAAGGTTTGGAGTATG
	*IL-10*	F-GTGATGCCCCAAGCTGAGA R-CACGGCCTTGCTCTTGTTTT
	*TNF-α*	F-CTGCTGCACT TTGGAGTGAT R-AGATGATCTG ACTGCCTGGG
Housekeeping gene	*ACTB* (β-actin)	F-ATTGCCGACAGGATGCAGAA R-GCTGATCCACATCTGCTGGAA
	*GAPDH* (Glyceraldehyde-3-phosphatedehydrogenase)	F-CAACAGCGACACCCACTCCT R-CACCCTGTTGCTGTAGCCAAA

### RNA extraction and quantification

Total RNA was extracted with MN NucleoSpin® RNA Plus KIT (Macherey–Nagel) according to the instructions of manufacturer. Briefly, the cell was added with 350 µl of lysis buffer before transferring to a column fixed in the collection tube and centrifuged at 11,000×*g* for 30 s. Then, 100 μL of binding solution was added to the column and centrifuged at 11,000×*g* for 15 s. The cell pellet was washed thrice with 400 μl of washing buffer. Wash solutions were removed by centrifugation at 11,000×*g* for 15 s and dried by centrifugation at 11,000×*g* for 2 min. Purified RNA was eluted by adding 30 μL RNase-free H_2_O and collected by centrifugation at 11,000×*g* for 1 min. The quality of the extracted RNA was determined by using BioSpectrometer kinetic (Eppendorf, Hamburg, Germany). The purity of RNA was determined from the adsorption ratio of 260/280. The extracted RNA that achieved more than 2.00 for the adsorption ratio of 260/280 was used for subsequent procedure.

### Reverse transcription (RT) and pre-amplification of cDNA

cDNA was prepared from the extracted RNA by reverse transcription using 5 µL Reverse Transcription Master mix (Fluidigm, California, USA). The resulted cDNA was then pre-amplified by using PreAmp Master mix (Fluidigm) with 7 custom-designed primers (designed by using Delta Gene assays according to the manufacturer's instructions). The pre-amplification reactions were performed with BioRad MyCycler Thermal Cycler (Bio-Rad Laboratories, Inc.) using the following amplification conditions: 95 °C for 2 min, followed by 14 cycles of 95 °C for 15 s and 60 °C for 4 min.

### Inflammation gene expression analysis

Gene expression analysis was performed with BioMark software (Fluidigm, California, USA). Quantitative PCR reaction were carried out in a Flex Six™ Genotyping IFC chip (Fluidigm, CA, USA). RT‑qPCR was performed on a Biomark HD system (Fluidigm, California, USA) using the following thermocycling conditions: 70 °C for 2400 s, 60 °C for 30 s, 95 °C for 60 s, followed by 30 cycles of 96 °C for 5 s and 60 °C for 20 s. The relative quantification method of 2^−ΔΔct^ of Fluidigm real-time PCR software Version 4.3.1 (Fluidigm, California, USA) was employed for the gene expression data analyses.

### Statistical analysis

Analysis of variance (ANOVA) was used for the data analyses in this study and the comparison of means was determined by using Duncan’s multiple range tests of SPSS version 22 (SPSS, Inc) at *p* ≤ 0.05.

## Data Availability

The datasets used and/or analysed during this study are available from the corresponding authors on reasonable request.

## Abbreviations

COX-2: Cyclooxygenase-2
*E. coli*: *Escherichia coli*
FFA3: Free fatty acid receptor 3
HDACs: Histone deacetylases
IC50: Inhibition concentration of 50% growth
IL: Interleukins
LPS: Lipopolysaccharide
NO: Nitric oxide
ORF: Open reading frame
SCFA: Short chain fatty acid(s)
TNF-α: Tumor necrosis factor α
